# Malaria and other febrile diseases among travellers: the experience of a reference centre located outside the Brazilian Amazon Region

**DOI:** 10.1186/s12936-016-1347-x

**Published:** 2016-05-26

**Authors:** Andréa Beltrami Dotrário, Lucas José Bazzo Menon, Valdes Roberto Bollela, Roberto Martinez, Daniel Cardoso de Almeida e Araújo, Benedito Antônio Lopes da Fonseca, Rodrigo de C. Santana

**Affiliations:** Departamento de Clínica Médica, Faculdade de Medicina de Ribeirão Preto, Universidade de São Paulo (USP), Ribeirão Preto, São Paulo 14049-900 Brazil; Departamento de Medicina Social, Faculdade de Medicina de Ribeirão Preto, Universidade de São Paulo (USP), Ribeirão Preto, São Paulo Brazil

**Keywords:** Malaria, Diagnosis, Acute febrile illness, Brazilian Amazon, *Plasmodiumfalciparum*, *Plasmodium vivax*

## Abstract

**Background:**

Malaria is endemic in countries located in tropical and sub-tropical regions. The increasing flow of domestic and international travellers has made malaria a relevant health problem even in non-endemic regions. Malaria has been described as the main diagnosis among travellers presenting febrile diseases after returning from tropical countries. In Brazil, malaria transmission occurs mainly in the Amazon region. Outside this area, malaria transmission is of low magnitude.

**Methods:**

This cross-sectional study aimed to describe the experience in the diagnosis of malaria in a reference centre located outside the Brazilian Amazon Region, emphasizing the differences in clinical and laboratory markers between cases of malaria and those of other febrile diseases (OFD). Medical charts from adult patients (≥18 years) who underwent a thick smear test (TST) for malaria, between January 2001 and December 2014, were retrospectively reviewed.

**Results:**

A total of 458 cases referred to perform the TST were included. Malaria was diagnosed in 193 (42 %) episodes. The remaining 265 episodes (58 %) were grouped as OFD. The majority of malaria episodes were acquired in the Brazilian Amazon Region. The median time between the onset of symptoms and the TST was 7 days. Only 53 (11.5 %) episodes were tested within the first 48 h after symptom onset. Comparing malaria with OFD, jaundice, nausea, vomiting, and reports of fever were more prevalent in the malaria group. Low platelet count and elevated bilirubin levels were also related to the diagnosis of malaria.

**Conclusions:**

The results indicate that outside the endemic area travellers presenting febrile disease suspected of being malaria underwent diagnostic test after considerable delay. The reporting of fever combined with a recent visit to an endemic area should promptly evoke the hypothesis of malaria. In these cases, specific diagnostic tests for malaria should be a priority. For cases that jump this step, the presence of elevated bilirubin or thrombocytopaenia should also indicate a diagnosis of malaria.

## Background

Malaria is endemic in 97 countries located in tropical and sub-tropical regions, where an estimated 3.3 billion people are at risk of infection [1]. The increasing flow of domestic and international travellers has made malaria a relevant health problem even in regions that are not endemic for the disease. Malaria is one of the main diagnoses among patients presenting febrile diseases after returning from tropical countries [2].

In the Americas, malaria occurs predominately in South America, where more than 70 % of cases are from Brazil, Venezuela and Colombia [1]. In Brazil, malaria transmission occurs predominantly in the Amazon Basin, which covers nine states: seven located in the Northern (Amapá, Acre, Amazonas, Pará, Rondônia, Roraima e Tocantins), one in the Center-West (Mato Grosso) and one in Northeastern (Maranhão) areas of the country [3, 4]. Outside this region, malaria transmission is of low magnitude and occurs in isolated foci. According to data from the Secretary of Health Surveillance of the Ministry of Health of Brazil, in 2014 approximately 139,000 autochthonous malaria cases were recorded in the country. Only 54 cases with probable autochthonous transmission were reported outside the Amazon region, which represents 0.03 % of the malaria transmission cases in the country for that year [5].

The case fatality rate was several times higher outside the Amazon region (2.68 %) than that found within the Amazon (0.02 %) in 2014 [5]. People living outside areas that are endemic for malaria are more susceptible to severe forms of the disease. This can be due to lack of specific immune response against *Plasmodium*, which depends on prolonged and repeated antigenic exposure [6]. Another factor to be considered is the risk of delay in diagnosis, as outside endemic areas physicians are less familiar with the management of malaria.

In this context, the present study aimed to describe the epidemiological, clinical and laboratory profiles of cases referred for malaria diagnosis, in a reference centre located outside the Brazilian Amazon Region. Another aim of the study was to identify differences in clinical and laboratory markers between cases of malaria and those of other febrile diseases (OFD).

## Methods

### Study design, subjects and setting

This cross-sectional study was conducted at the University Hospital of the Ribeirão Preto Medical School, University of São Paulo. It is located in the city of Ribeirão Preto, in the northwest region of São Paulo State, in southeastern Brazil (21º10′40″S, 47º48′36″W). The hospital is one of fourteen reference centres of malaria diagnosis and treatment located in this state and covers 90 municipalities. Patients suspected of having malaria are referred to the centre to perform a thick smear test (TST). Eventually the patients spontaneously seek the hospital for care. Diagnostic tests other than TST were not routinely performed over the period analysed. Medical charts from adult patients who underwent a TST, between January 2001 and December 2014, were retrospectively reviewed.

Adult patients (≥18 years) referred due to an acute illness, who performed the TST for diagnostic purposes, were included in the study. Those cases in which the TST was performed to monitor parasitaemia after malaria chemoprophylaxis or treatment were excluded. Cases of relapse, defined as new onset of symptoms after treatment in the study centre and without history of further exposure in risk areas, were not included. For these cases, only the first malaria episode was included. However, a new onset of symptoms in a patient who had been appropriately treated for malaria and was subjected to new exposure in a risk area was considered a new case and was included.

Standardized forms were used for data collection from the medical charts. Demographic data, signs and symptoms, time between onset of symptoms and diagnosis, travel history, laboratory results (complete blood count test, total bilirubin levels, serum creatinine, alanine aminotransferase, aspartate aminotransferase, and TST) and chest radiograph performed up to 72 h from the first attendance, were registered. For clinical data, only records with precise information about the presence or absence of the required sign or symptom were considered.

Properly trained professionals performed the TST during the first attendance, within the first hour after blood sample collection. Each test was independently reviewed by another professional.

### Ethical aspects

Data were obtained from medical charts. We have not obtained informed consent from participants. The study protocol and informed consent waiver was approved by the Research Ethics Committee of HCFMRP-USP (number 13388/2014).

### Diagnostic definitions and malaria severity criteria

Malaria diagnosis was defined by visual detection of *Plasmodium* species in the peripheral blood by TST. Patients were divided into two groups according to the diagnostic definition:

- Malaria group: patients with a confirmed diagnosis of malaria.
- Other febrile diseases (OFD): patients with acute disease not diagnosed as malaria.

According to the criteria of the World Health Organization (WHO) [7], severe malaria was defined as the presence of at least one of the following findings: impaired consciousness, circulatory collapse, acute renal failure (serum creatinine >3 mg/dL), severe anaemia (haemoglobin <7 g/dL), jaundice associated with vital organ dysfunction, pulmonary oedema, high parasitaemia (>5 %) or death.

### Statistical analysis

Statistic analysis was performed using SAS software (SAS system for Windows, version 9.2. SAS Inst, Cary, NC, USA). Continuous variables were expressed as median and interquartile range (IR). In order to compare the continuous variables between the two groups (malaria and ODF) the Mann–Whitney test was used. Qualitative variables were compared using the Chi square test. All tests were two-tailed. A p value <.05 was considered significant.

## Results

### Characteristics of the subjects

Over the period analysed, 613 TST performances were registered. Of these episodes, 458 were included in the study, which related to 443 patients. The remaining 155 episodes were excluded due to incomplete clinical information and lab data (n = 37), the subject being under 18 years of age (n = 34), cases presenting chronic fever in the inpatient scenario (n = 37), the subject being asymptomatic (n = 26), prolonged fever in subjects infected with HIV (n = 3) and cases of malaria relapse (n = 18). The majority of the patients included were male (n = 380; 83 %) with a median age of 39 years. The majority of the subjects were in the age group 18–50 years (n = 345; 75 %). Of the 458 episodes evaluated, 193 (42 %) produced a confirmed diagnosis of malaria. For the remaining 265 episodes (58 %), not diagnosed as malaria, a syndromic or aetiological diagnosis was defined in only 83 of them (Table 1). The median time between the onset of symptoms and the TST was 7 days for the sample as a whole. Only 53 (11.5 %) episodes were tested within the first 48 h after symptom onset. Specifically considering the 193 malaria cases, only 21 cases were tested within this period (10.8 %).

**Table 1:** Diagnostic condition defined in 83 non-malaria cases

Diagnosis	Number
Visceral leishmaniasis	2
Aseptic meningitis	2
Acute diarrhea	9
Urinary tract infection	5
Dental infection	2
Acute pneumonia	8
Intra-abdominal abscess	1
Dengue	12
Common cold	5
Cholangitis	2
Spondylodiscitis	1
Leptospirosis	2
Infective endocarditis	2
Hanseniasis	1
Pharyngotonsillitis	3
Acute sinusitis	2
Cellulitis	1
Liver abscess	1
Acute toxoplasmosis	4
Histoplasmosis	3
Acute HIV infection	1
Brucellosis	1
Infectious mononucleosis	1
Hantaviruses	1
Renal lithiasis	3
Drug hypersensitivity	1
Erythema nodosum	2
Postoperative fever	1
Glomerulonephritis	1
Malignancy	2
Alcoholic hepatitis	1

### Clinical, epidemiological and laboratory characteristics of the malaria cases

**Fig. 1:**
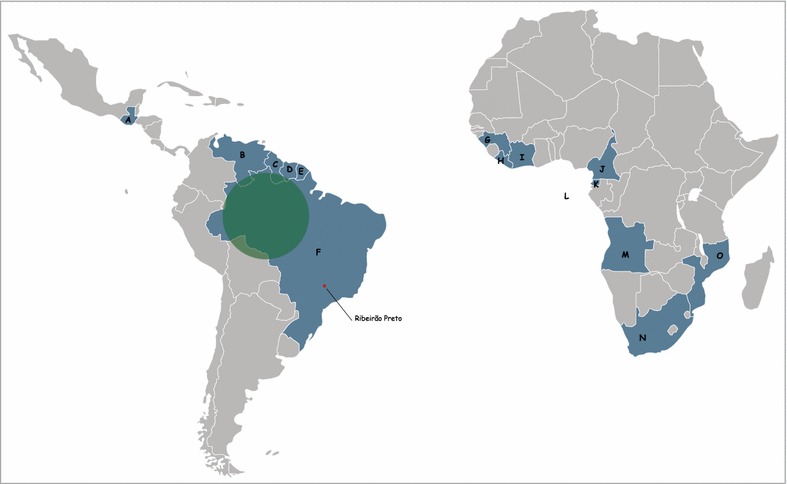
Regions of malaria infection. *a* Guatemala; *b* Venezuela; *c* Guyana; *d* Suriname; *e* French Guiana; *f* Brazil; *g* Guinea; *h* Liberia; *i* Côte d’Ivoire; *j* Cameroon; *k* Equatorial Guinea; *l* São Tomé and Principe; *m* Angola; *n* South Africa; *o* Mozambique. Cases from Brazil originated in the Amazon region (*green area*). Ribeirão Preto (study site location) is represented by the *red dot*

Of the 193 malaria cases, the majority occurred in men, with a median age of 37 years. Most subjects in this group also belonged to the 18–50 years age group (n = 154; 79 %). With regard to the probable region of malaria infection, 73.5 % (n = 142) of the cases occurred in subjects who had recently visited a Brazilian state located in the Amazon region. All these states represent risk areas for malaria, according to data from the Ministry of Health of Brazil. The remaining 51 cases reported recent travel to other countries: 18 cases had visited South America (Guiana, French Guiana, Venezuela, Suriname) and Central America (Guatemala) and, in 33 cases, the subjects had recently been to countries of western and sub-Saharan Africa (Angola, Mozambique, South Africa, Guinea, Equatorial Guinea, Liberia, Cameroon, Ivory Coast, São Tomé and Príncipe). Figure 1 shows the probable geographic sites of malaria infection.

The distribution of *Plasmodium* species involved and the sites of infection are shown in Table 2. There was a predominance of malaria caused by *Plasmodium vivax* (n = 132; 68.4 %) followed by *Plasmodium falciparum* (n = 45; 23.3 %). Mixed infections with *P. vivax* and *P. falciparum* were detected in 15 episodes (7.8 %). Only one case involving *Plasmodium malariae* was diagnosed. The majority of vivax malaria cases (n = 115; 87 %) were probably acquired in the Brazilian Amazon Region. Of the 45 falciparum malaria episodes, 27 were acquired in African countries. Severe malaria occurred in seven cases (4 %). One of them evolved to death. Four infections of *P. falciparum* (9 %) and three of *P. vivax* (2 %) were classified as severe. All seven of these cases were hospitalized. Three falciparum malaria cases were treated with intravenous artesunate and the other one with oral quinine sulfate plus tetracycline. All three vivax malaria episodes were treated with oral chloroquine plus primaquine.

**Table 2:** *Plasmodium* species according to the site of infection

Country	State	*Plasmodium* species (n)				
		*Pv*	*Pf*	*Pv* + *Pf*	*Pm*	Total
Brazil		115	17	9	1	142
	Rondônia	39	7	4	0	50
	Pará	33^a^	6^a^	1	0	40
	Amazonas	11	1	1	1	14
	Maranhão	10	0	2	0	12
	Mato Grosso	8^a^	1	1	0	10
	Acre	7	1	0	0	8
	Roraima	3	1	0	0	4
	Amapá	3	0	0	0	3
	Tocantins	1	0	0	0	1
Angola		2	10^a^	1	0	13
Guiana		6	0	3	0	9
Mozambique		0	9^b^	0	0	9
French Guiana		4	1	1	0	6
South Africa		1	4^a^	0	0	5
Guinea		0	1	0	0	1
Guatemala		1	0	0	0	1
Equatorial Guinea		1	0	0	0	1
Venezuela		1	0	0	0	1
Ivory Coast		0	1	0	0	1
Cameroon		0	0	1	0	1
Liberia		0	1	0	0	1
São Tomé and Príncipe		0	1	0	0	1
Suriname		1	0	0	0	1
Total		132	45	15	1	193

*Pv*, *Plasmodium vivax*; *Pf*, *Plasmodium falciparum*; *Pm*, *Plasmodium malariae*

^a^Represents a severe case

^b^Represents a fatal case

**Fig. 2:**
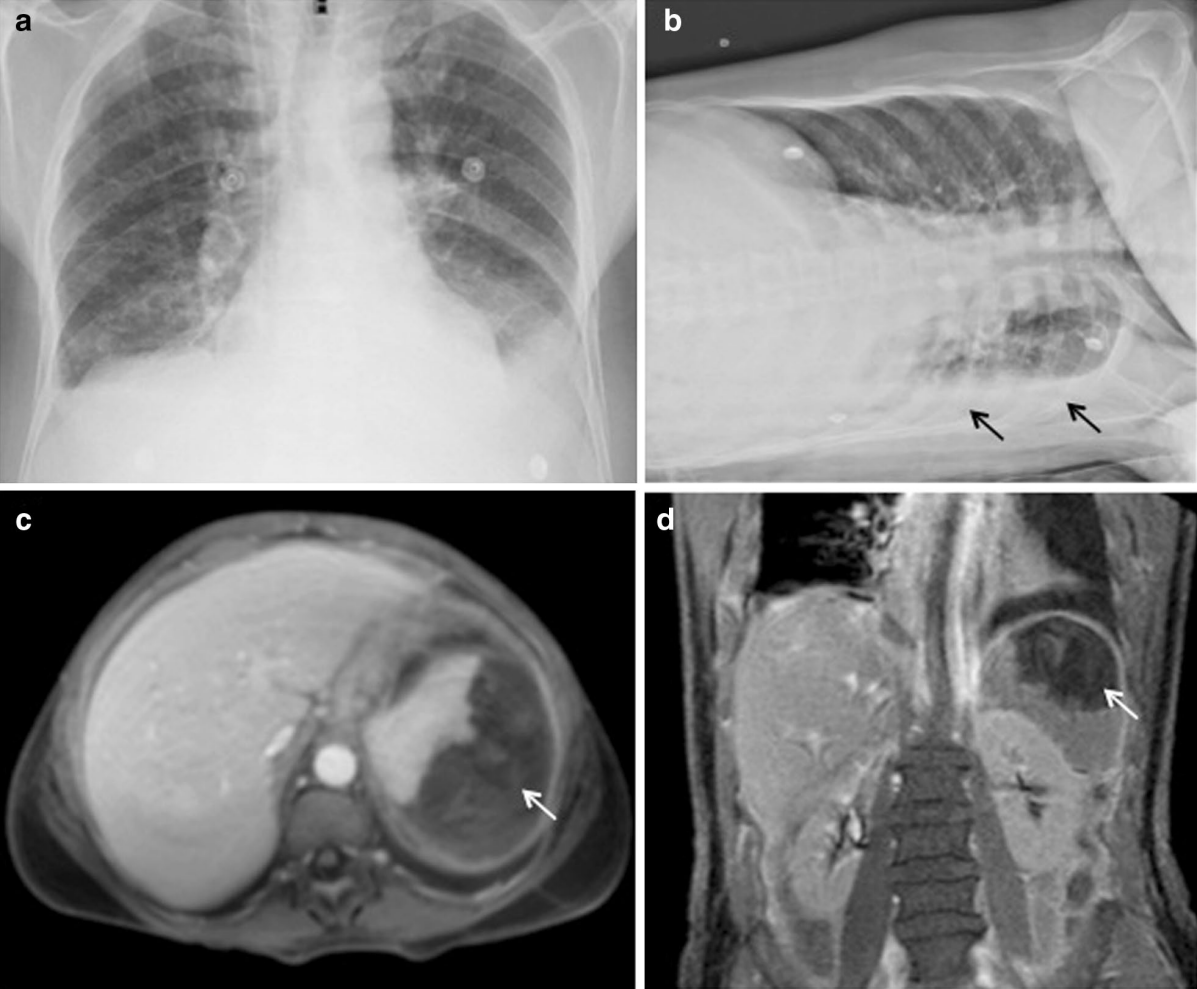
Radiologic findings of a man, 60 years of age, presenting severe vivax malaria. **a** Chest radiograph (PA view) showing interstitial opacities in both lungs compatible with pulmonary congestion. Obliteration of the costphrenic angles due to small bilateral pleural effusion can also be observed. **b** In the left lateral decubitus the pleural effusion is seen as fluid layering along the rib cage (*black arrows*). **c** Abdominal magnetic resonance imaging (T1-weighted axial image) reveals spontaneous sub-capsular splenic haematoma (*white arrow*). **d** The splenic haematoma is shown as T1-weighted coronal MRI image (*white arrow*)

A complete blood count test was available in 169 out of the 193 malaria episodes. Severe anaemia, defined when haemoglobin levels were <7 g/dL, was present in only one episode. However, a significant reduction in haemoglobin levels (7–10 g/dL) was observed in 19 cases (11 %), of which 14 were due to vivax malaria. Severe thrombocytopaenia (<50,000/μL) was detected in 34 cases (20 %): 17 due to *P. vivax*, ten to *P. falciparum* and seven to mixed infections of *P. vivax* and *P. falciparum*. No serious bleeding episode was detected among these cases. In one malaria vivax episode, in which the platelet count was 54,000/μL, a spontaneous sub-capsular splenic haematoma was diagnosed (Fig. 2). The same patient presented hypoalbuminaemia (serum albumin = 1.9 g/dL) associated with peripheral oedema and pleural effusion, which resolved after anti-malarial treatment. The majority of the malaria episodes were treated in an outpatient setting. However, a considerable portion of malaria patients were hospitalized after diagnosis (n = 85; 44 %).

### Clinical and epidemiological characteristics of non-malaria cases

In this group, composed of 265 episodes, the majority of the individuals were also male (n = 212; 80 %). The median age of the subjects in this group was 41 years. In 182 cases (68.6 %), a specific diagnosis was not defined during the follow-up period. The majority of the cases were treated in an outpatient setting (n = 197; 74.3 %), among which 103 (52 %) did not attend the scheduled return visits after initial presentation. Regarding travel history, the majority of the patients in this group reported a recent visit to a Brazilian state (n = 230; 86.7 %), especially those located in the Amazon basin. Sixty-two (27 %) cases in this group had visited states located outside the Amazon region, of which 21 (9 %) reported travel to areas that are not considered at risk for malaria transmission.

### Comparison of clinical and laboratory characteristics between malaria and OFD cases

The clinical characteristics of the subjects diagnosed with malaria and OFD are shown in Table 3. Malaria cases had TST performed earlier. Jaundice, nausea, vomiting and reports of fever in the 48 h prior to presentation were more prevalent in the malaria group. The results of the complementary examinations are shown in Table 4 for both groups. Low platelet count and elevated bilirubin levels were the main laboratory findings related to malaria. A chest radiograph was more often requested in OFD cases than in those of malaria (24.5 vs 12.4 %; p < .001). Furthermore, malaria cases were more frequently hospitalized than OFD cases (44 vs 26 %; p < .001). Considering the non-malaria cases for which a diagnosis was defined, dengue fever was the most frequent (12/83; 14 %). Compared to the malaria (data not shown in the table), dengue fever was related to lower median levels of white blood cells [3300/μL vs 5400/μL; p < .001; (dengue n = 11 vs malaria n = 171)] and higher hemoglobin levels [14.5 vs 12.7 g/dL; p = .002; (dengue n = 11 vs malaria n = 170)]. Malaria was related to higher median total bilirubin levels [1.45 vs 0.43 mg/dL; p < .001; (dengue n = 11 vs malaria n = 146)]. Although the median platelet count was lower in malaria cases [84.5 × 10^3^/μL; (n = 168)] than those of dengue fever [113 × 10^3^/μL; (n = 11)], this difference was not significant (p = .06).

**Table 3:** Demographic and clinical characteristics of malaria and other febrile diseases

Variable	Malaria	OFD	p value
Age (years) [median (IQR)]	37 (29.0–48.0)	41 (31.0–52.0)	.03
Males [n (%)]	167 (86.5)	212 (80)	NS
Interval from presentation to TST (days) [median (IQR)]	6 (3.0–10.0)	7 (4.0–15.0)	.04
Chills [proportion^a^ (%)]	118/124 (95.2)	115/125 (92.0)	NS
Myalgia [proportion^a^ (%)]	113/120 (94.2)	148/166 (89.2)	NS
Jaundice [proportion^a^ (%)]	41/175 (23.4)	27/222 (12.2)	<.01
Cough [proportion^a^ (%)]	27/100 (27.0)	63/154 (40.9)	.02
Dyspnea [proportion^a^ (%)]	15/105 (14.3)	15/109 (13.8)	NS
Nasal discharge [proportion^a^ (%)]	3/36 (8.3)	16/55 (29.1)	.02
Nasal obstruction [proportion^a^ (%)]	5/37 (13.5)	12/43 (27.9)	NS
Chest pain [proportion^a^ (%)]	2/40 (5)	15/65 (23.1)	.01
Cervical lymphadenopathy [proportion^a^ (%)]	4/57 (7)	24/88 (27.3)	<.01
Nausea [proportion^a^ (%)]	58/112 (51.8)	42/114 (36.8)	.02
Vomiting [proportion^a^ (%)]	60/136 (44.1)	31/129 (24.0)	<.01
Diarrhea [proportion^a^ (%)]	22/136 (16.2)	52/157 (33.1)	<.01
Sore throat [proportion^a^ (%)]	4/43 (9.3)	15/53 (28.3)	.02
Abdominal pain [proportion^a^ (%)]	43/72 (59.7)	48/82 (58.5)	NS
Headache [proportion^a^ (%)]	120/138 (87.0)	137/166 (82.5)	NS
Retro-orbital pain [proportion^a^ (%)]	25/40 (62.5)	30/46 (65.2)	NS
Hepatomegaly [proportion^a^ (%)]	35/184 (19.0)	38/327 (16.0)	NS
Splenomegaly [proportion^a^ (%)]	20/183 (10.9)	18/237 (7.6)	NS
Fever^b^ [proportion^a^ (%)]	193/193 (100 %)	242/258 (93.8)	<.01

p value <.05 was considered statistically significant

Mann–Whitney test was used to compare continuous variables between the groups

Chi square test was used to compare categorical variables between the groups

*OFD* other febrile diseases, *IQR* interquartile range, *TST* thick smear test

^a^Proportion of positive results of the total cases where the variable was accurately recorded

^b^Report of fever (symptom)

**Table 4:** Laboratory findings of malaria and other febrile diseases (OFD)

Variable [median (IQR)]	Malaria	OFD	p value
Hemoglobin (g/dL)^a^	12.7 (11.5–14.2)	13.9 (12.8–15.0)	<.01
Hematocrit (%)^a^	38.8 (34.0–42.0)	42.0 (38.2–45.0)	<.01
WBC (μL)^a^	5400.0 (4300.0–6900.0)	6650.0 (4700.0–8900.0)	<.01
Platelet × 10^3^ (μL)^a^	84.5 (55.5–131.0)	204.0 (148.0–269.0)	<.01
AST (U/L)^b^	31.5 (23.0–48.0)	32.0 (22.8–51.0)	NS
ALT (U/L)^c^	43.6 (28.0–60.0)	49.5 (30.0–89.0)	NS
Total Bilirubin (mg/dL)^d^	1.45 (0.96–2.27)	0.58 (0.42–1.03)	<.01
Serum creatinine (mg/dL)^e^	1.0 (0.9–1.2)	1.0 (0.9–1.2)	NS

p value <.05 was considered statistically significant

Mann–Whitney test was used to compare variables between the groups

*OFD* other febrile diseases *IQR* interquartile range, *WBC* white blood cells, *NS* not significant

^a^Performed in 169 malaria and 210 OFD cases

^b^Performed in 163 malaria and 184 OFD cases

^c^Performed in 66 malaria and 87 OFD cases

^d^Performed in 145 malaria and 157 OFD cases

^e^Performed in 157 malaria and 172 OFD cases

## Discussion

This study was conducted in a university hospital located in Southeastern Brazil, in a state where malaria transmission is infrequent and occurs in isolated areas. As expected, the malaria cases diagnosed reflected, for the most part, the endemicity of the Brazilian Amazon. None of the cases acquired in Brazil had a state located outside the Amazon region as the probable site of infection. Episodes that originated in Africa contributed to 60 % of the total *P. falciparum* infections. These cases were responsible for the higher prevalence of falciparum malaria (23 %) found in this study compared to that described for the Amazon region in 2014 (16 %) [5].

It was found that in 9 % of the non-malaria episodes referred to perform TST, all originating in Brazil, the subjects reported that they had travelled to regions of the country considered to be without risk for the transmission of malaria. This indicates that a considerable number of general practitioners lack knowledge regarding malaria epidemiology or have excessive concern with the description of sporadic autochthonous cases occurring outside of the Amazon region. Recently, an increase in the frequency of malaria episodes was described in specific locations outside the Amazon region. In Goiânia City, located in Goiás State, central-west Brazil, between October 2014 and March 2015, malaria transmission was described in a forested area located in the centre of the city. In the State of Rio de Janeiro, outside the city of Rio de Janeiro, the incidence of vivax malaria has been reported to be higher than the annual average of six autochthonous cases. Between January and February 2015, 23 malaria cases were reported in regions covered by Atlantic Forest [8]. Travellers suspected of having malaria were referred for diagnostic testing after a considerable delay. The mean interval between the onset of symptoms and the diagnostic test was 7 days for all cases and 6 days considering only those diagnosed as malaria. Only 11.5 % of the cases had a TST performed in the first 48 h of symptoms. This differs from the practice in the Amazon region, where 56.4 % of malaria cases, in 2011, were diagnosed and treated within the first 48 h of symptom onset [9]. The delay observed in this study indicates a low index of suspicion for malaria by physicians working outside the endemic areas. In reference centres located in the Amazon region, the TST is a priority test for patients presenting acute febrile disease.

Despite this delay in completing the diagnostic test, only seven severe malaria episodes (4 %) were found and there was only one death during the 14-year period evaluated in the study. In a university hospital with characteristics similar to that of the study centre, located in the city of Campinas, also in São Paulo State, there were no cases of death among 263 malaria cases treated between 1998 and 2011. In that study, the authors also found a mean interval between the onset of symptoms and the diagnosis of 6 days for infections caused by *P. vivax*. However, this interval was slightly lower (4 days) for falciparum malaria episodes [10].

The low percentage of severe malaria found in the present study may be partly explained by the prevalence of non-falciparum malaria episodes. This is a reflection of the distribution of *Plasmodium* species in Brazil. According to the latest bulletin of the Ministry of Health, *P. vivax* was responsible for 84 % of the reported malaria episodes, while *P. falciparum* occurred in only 16 % of them (5). In the USA, where almost 60 % of malaria cases among travellers in 2012, were caused by *P. falciparum*, 14 % of the episodes were classified as severe. The majority of the severe cases (75 %) were due to *P. falciparum* [11]. In this same study, considering only individuals aged ≥18 years, the percentage of severe cases (11 %) was still greater than that verified in the present study. Data related to imported cases of malaria in the UK, between 1987 and 2006, indicate that the case fatality rate among falciparum malaria episodes (0.73 %) was 15 times greater than those of non-falciparum malaria (0.05 %) [12]. The non-inclusion of children, a risk group for severe malaria, may also have contributed to the low percentage of severe cases found in the present study. Mascarello et al. [13], in a study conducted on imported malaria infections in Italy, found 4.5 % severe malaria episodes among adults, compared to 18.6 % among subjects younger than 15 years.

In spite of the delay in the referral to perform a TST, it can be assumed that, for the majority of the subjects tested, diagnosis and treatment was completed within a period sufficient to prevent the onset of complications. This is not only supported by the low percentage of severe forms, but also by the evaluation of the laboratory results of the malaria cases at the moment of diagnosis. The mean haemoglobin and serum creatinine values were normal, with bilirubin levels and platelets count presenting mild abnormalities. However, a number of patients presented relevant laboratory abnormalities at the moment of evaluation. Moderate anaemia (haemoglobin = 7–10 g/dL) and severe thrombocytopaenia (<50,000/μL) were detected in 11.3 and 20 %, respectively. Although these abnormalities are not markers of severe malaria, they can increase severity in the presence of co-morbidities [14].

The low mortality and severity rates observed in this study may be underestimated due to selection bias. Cases in which malaria was not suspected may not have been referred to the study centre. The fatality rate of cases reported outside the Brazilian Amazon Region is several times higher than that described within the Amazon. Between 2000 and 2011, this indicator ranged from 0.04 to 0.02 % and from 0.6 to 1.8 % within and outside the Amazon region, respectively [9]. A low index of diagnostic suspicion for malaria by physicians working outside the endemic areas is the main reason for this scenario. This assumption is supported by findings observed in other regions of Brazil [15] and other countries [12, 16], which relate a delay in diagnosis to malaria mortality in non-endemic areas. Considering that malaria prevalence has decreased globally over the past years [1], this could be particularly concerning. As in some areas where malaria has become progressively less common, clinicians will have less experience in making diagnosis.

Three severe vivax malaria episodes were detected. Furthermore, infections due to *P. vivax* were responsible for 73.6 % of the moderate anaemia cases and 50 % of severe thrombocytopaenia. One of these cases presented severe hypoalbuminaemia associated with peripheral oedema, pleural effusion and pulmonary congestion.

Comparing malaria with OFD, the detection of jaundice and the report of nausea, vomiting and fever were more prevalent in the patients with malaria. Fever has been reported in at least 90 % of subjects diagnosed with malaria and is the main reason for seeking care among travellers with malaria, according to different studies [2, 17–19]. Although fever is an important clue for the diagnosis of malaria, some considerations need to be made. Despite fever being frequently reported, temperature ≥38.0 °C is not detected in a considerable proportion of patients at the moment of diagnosis [18]. Furthermore, as fever is the main reason why travellers seek medical evaluation, the high prevalence of this symptom in several studies can be associated with reference bias. Absence of fever can be observed more frequently in subjects undergoing chemoprophylaxis [20] and in semi-immune individuals, which is seen more among inhabitants of endemic areas [21, 22]. Nausea and vomiting have been reported in 13–45 % of imported malaria cases [16, 18, 23, 24]. Nonetheless, these symptoms are too unspecific to be used as clinical predictors of malaria [25]. Thus, they must be recognized as possible clinical manifestations of malaria and should not devalue this diagnosis in favour of diseases in which the symptoms are predominantly gastrointestinal, such as acute gastroenteritis, viral hepatitis and typhoid fever, which are also commonly diagnosed among travellers returning from the tropics [2, 26].

In relation to clinical aspects, a higher prevalence of upper airway symptoms, such as cough, nasal discharge and sore throat, was found among the non-malaria cases. Cervical adenopathy and diarrhoea were also more prevalent in this group. These signs and symptoms suggest that viral or bacterial diseases of the upper airways and gastrointestinal tract probably occurred in a considerable number of the non-malaria cases. In this group, upper airway and gastrointestinal infections, taken together, occurred in a third of the cases in which a diagnosis was defined. This finding is supported by other studies, indicating that upper respiratory tract infections and gastroenteritis are the most common diagnoses after malaria among travellers returning from tropical countries [2, 25, 27]. Whereas these infections usually have a benign and self-limiting clinical course, this may explain the finding that the majority of non-malaria cases were monitored in an outpatient setting and that the individuals did not attend scheduled return visits.

In addition to upper airway symptoms being more prevalent in the non-malaria cases, reports were detected of sore throat and nasal symptoms in 8.3–13.5 % of the malaria cases. A study conducted in the Brazilian Amazon Region described the presence of nasal discharge, sneezing and sore throat in 18.1–22.2 % of patients diagnosed with malaria [22]. The hypothesis that concomitant upper respiratory tract viral infections were responsible entirely or partly for these symptoms cannot be discounted. Thus, even though these complaints are suggestive of common cold or influenza, upper airway symptoms described are associated with malaria.

In the present study, splenomegaly was not predictive of malaria and was reported in only 11 % of the cases. This percentage is considerably lower than that reported in other studies, which describe the presence of splenomegaly in 23–33 % of cases among travellers [19, 28, 29]. This difference may be due to the retrospective nature of this study. A high under-reporting of signs and symptoms was observed. The lack of detection during the physical examination due to negligence or inexperience of the attendant physician should also be considered. A careful physical examination is important since splenomegaly has been shown to be the most important clinical predictor of malaria [30].

With regard to laboratory tests, elevated bilirubin levels and thrombocytopaenia were the most prominent results related to the diagnosis of malaria. Although reductions in haemoglobin, haematocrit and white blood cell count were also more prevalent in the malaria cases, these abnormalities were not as pronounced as the reduced platelet count. These results are in agreement with the results from other studies, which highlight hyperbilirubinaemia (total bilirubin >1.2 mg/dL) and thrombocytopaenia (platelet count <150 × 10^3^/μL) as the main laboratory predictors of malaria [25, 29, 30]. The use of the platelet count as a predictor of malaria can be particularly problematic in travellers returning from regions where dengue fever is also endemic, such as Brazil. Both are characterized by fever of acute onset and may present with similar abnormalities in the blood count tests, such as thrombocytopenia and leukopaenia, as well as elevations in aminotransferase levels. The presence of a rash, unusual in malaria, normal levels of C-reactive protein and leukopaenia has been described more related to dengue fever than to malaria [31, 32]. In the present study it was also detected that dengue fever is more probable in subjects with normal total bilirubin levels and without reduced hemoglobin levels.

The present study has significant limitations, some having been mentioned previously. It should be noted that a clear selection bias is defined, considering that symptomatic patients were predominantly referred to the study centre. Due to the retrospective nature of the study, precise data on some variables included were not accurately registered, generating a considerable amount of missing data. Furthermore, the malaria diagnosis was defined exclusively through the use of the TST. The use of more sensitive methods, such as PCR, enables diagnoses in cases of infection with low parasitaemia, in which TST may provide a negative result. This method also better defines whether cases classified as severe vivax malaria are in fact cases of concomitant infection with *P. falciparum.*

## Conclusions

The results of this study provided relevant information regarding the management of suspected cases of malaria, in a real life scenario, in a centre outside an endemic area. The results indicate that in non-endemic regions a large number of patients experience a delay in seeking or being referring to medical attention. This suggests that outside endemic areas physicians are less familiar with the diagnostic aspects of malaria. Continuing medical education on the diagnosis of malaria is necessary in these regions. The results also indicate that the reporting of fever combined with a recent visit to an endemic area should promptly evoke the hypothesis of malaria. In these cases, specific diagnostic tests for malaria should be a priority. For cases that ‘jump’ this step, the presence of elevated bilirubin or thrombocytopaenia should also indicate a diagnosis of malaria.
