# Correction to: Malaria and other febrile diseases among travellers: the experience of a reference centre located outside the Brazilian Amazon Region

**DOI:** 10.1186/s12936-018-2421-3

**Published:** 2018-07-30

**Authors:** Andréa Beltrami Doltario, Lucas José Bazzo Menon, Valdes Roberto Bollela, Roberto Martinez, Daniel Cardoso de Almeida e Araújo, Benedito Antônio Lopes da Fonseca, Rodrigo de C. Santana

**Affiliations:** 1 Departamento de Clínica Médica, Faculdade de Medicina de Ribeirão Preto, Universidade de São Paulo (USP), Ribeirão Preto, São Paulo 14049-900 Brazil; 2 Departamento de Medicina Social, Faculdade de Medicina de Ribeirão Preto, Universidade de São Paulo (USP), Ribeirão Preto, São Paulo Brazil

## Correction to: Malar J (2016) 15:294 10.1186/s12936-016-1347-x

Following publication of the original article [1], one of the authors flagged that unfortunately their last name, Doltario, was incorrectly spelled as ‘Dotrario’. This has since been corrected in the original article [1].
